# Effects of Nb Content on the Microstructure and Mechanical Properties of Deposited Metal in 960 MPa Grade Low-Alloy High-Strength Steel

**DOI:** 10.3390/ma19122647

**Published:** 2026-06-19

**Authors:** Xuan Liu, Shuqiang Jin, Feiyang Ji, Lihua Yu, Junhua Xu

**Affiliations:** School of Material and Science, Jiangsu University of Science and Technology, Zhenjiang 212000, China; shiya_404@163.com (X.L.); jinneey@163.com (S.J.); feiyang_ji@163.com (F.J.)

**Keywords:** low-alloy high-strength steel, deposited metal, microalloying element Nb, microstructure, mechanical properties

## Abstract

In this study, manual welding electrodes with varying niobium (Nb) contents (0, 0.05, and 0.1 wt%) were developed for 960 MPa grade low-alloy high-strength steel, and deposited metals were produced through multilayer multipass welding. Microstructural characterization and mechanical testing were performed using scanning electron microscopy (SEM), transmission electron microscopy (TEM), X-ray diffraction (XRD), electron backscatter diffraction (EBSD), and a universal testing machine to investigate the influence of Nb content and elucidate the strengthening mechanisms. The results demonstrate that under identical welding conditions, multipass thermal cycles induced a primary microstructural transformation from martensite to tempered martensite in all deposited metals, which predominantly comprised tempered martensite with minor fractions of bainite and second-phase particles. Increasing Nb content led to significant grain refinement. The second-phase particles exhibited sizes of 0.158 μm, 0.176 μm, and 0.168 μm, respectively, with volume fractions of 5.69%, 5.82%, and 5.90%. Nb addition substantially enhanced hardness and strength while causing a noticeable reduction in low-temperature impact toughness, though the values remained within acceptable limits. The deposited metal containing 0.05 wt% Nb exhibited optimal comprehensive mechanical properties, with a hardness of 386.7 HV, tensile strength of 1060 MPa, yield strength of 962 MPa, and Charpy impact energies of 41.95 J and 33.17 J at −40 °C and −60 °C, respectively. Theoretical calculations revealed that the dislocation strengthening contribution in martensite increased from 526 MPa to 600 MPa with increasing Nb content, representing the dominant strengthening mechanism, while grain refinement strengthening increased from 135.5 MPa to 157.6 MPa.

## 1. Introduction

The 960 MPa grade low-alloy high-strength (HSLA) steel, featuring high strength and good toughness, has found extensive applications in marine engineering, hydroelectric power generation, construction machinery, and other fields [1,2]. However, enhancing strength and toughness without compromising weldability has become a focal point of recent research [3]. Currently, microalloying (with elements such as Nb, Ti, V [4,5], and rare earth elements Sc, Y [6,7,8]) together with optimization of welding process parameters [9,10] constitutes the primary strategy for improving the microstructure and mechanical properties of welded joints. Among these approaches, microalloying represents a crucial method for enhancing HSLA steel performance, with the incorporation of trace niobium (Nb) having attracted considerable attention owing to its distinctive metallurgical effects.

The most prominent effect of niobium is the formation of dispersed carbide or carbonitride particles in steel, generating a grain boundary “pinning” effect that impedes austenite grain growth and recrystallization, thereby refining the microstructure. When these particles are dispersed within the ferrite matrix, they create strong obstacles to dislocation motion, leading to increased yield strength [11]. Hardy Mohrbacher et al. [12] observed in Nb-containing DP980 dual-phase steel that after annealing, the ferrite matrix contained dispersed NbC particles with an average diameter of approximately 5.3 nm and spacing of about 248 nm. Strengthening increments calculated via the Orowan mechanism for these precipitates reached as high as 180 MPa. Chen and Pan [11,13] asserted that the precipitation strengthening efficacy of Nb depends on the size and quantity of precipitates; different forms of Nb may lead to dissolution or coarsening of Nb(C, N) at elevated temperatures, and excessively large NbC precipitates would reduce strengthening efficiency and potentially impair toughness. Nb can induce either embrittlement or softening in the heat-affected zone (HAZ). Yang et al. [14] observed a softened HAZ band in Nb-containing high-strength steel, which was primarily attributed to Nb-promoted precipitation of acicular ferrite and tempered bainite, resulting in locally softer microstructures. Yu et al. [3] considered that M/A islands induced by Nb segregation may appear in the HAZ, forming hard and brittle zones. However, Wang et al. [15] argued that Nb can enhance austenite hardenability and promote the formation of high-strength bainite, thereby ameliorating hardness softening phenomena in the fine-grained HAZ (FGHAZ). Qi et al. [16] discovered through Nb addition in Ni-based welding consumables that Nb exerts strong precipitation strengthening effects, with intragranular precipitates being NbC and Ni_3_Nb phases, and grain boundary precipitates being NbC. Meanwhile, the increased area fraction of Nb-rich phases significantly reduced the low-temperature impact toughness but enhanced the tensile strength. Mohammed Ali et al. [17] believed that Nb-promoted precipitation of Al_2_O_3_, MnS, and complex inclusions was a critical factor in toughness degradation, as they readily serve as crack initiation sites. In summary, while numerous researchers have investigated the effects of Nb on the mechanical properties of HSLA steel and nickel-based alloys, the concurrent achievement of strength and toughness in the deposited metal of welded 960 MPa-grade HSLA steel remains challenging [18], thereby restricting its engineering applications. Furthermore, the influence of Nb content variation on the microstructure and mechanical properties of welding consumables remains scarcely investigated, and the underlying mechanisms of Nb in 960 MPa-grade high-strength deposited metals have yet to be fully elucidated.

In this study, three NiCrMo-alloyed deposited metals with varying niobium (Nb) contents were obtained by adjusting the Nb content in the electrode coating and employing the shielded metal arc welding (SMAW) process. The effects of different Nb contents on the microstructure, hardness, tensile strength, and low-temperature impact toughness of the deposited metals were investigated, aiming to achieve strength-matched properties with target values of 1020 MPa tensile strength, 960 MPa yield strength, and 27 J low-temperature impact toughness at −60 °C. This study achieves the synergistic optimization of strength and low-temperature impact toughness in 960 MPa-grade low-alloy high-strength steel, reveals the effect of Nb on the microstructural evolution and strengthening–toughening mechanisms of deposited metals under multilayer multipass welding conditions, and provides valuable guidance for the development of welding consumables for 960 MPa-grade low-alloy high-strength steels.

## 2. Experiments

### 2.1. Material Processing

**Figure 1:**
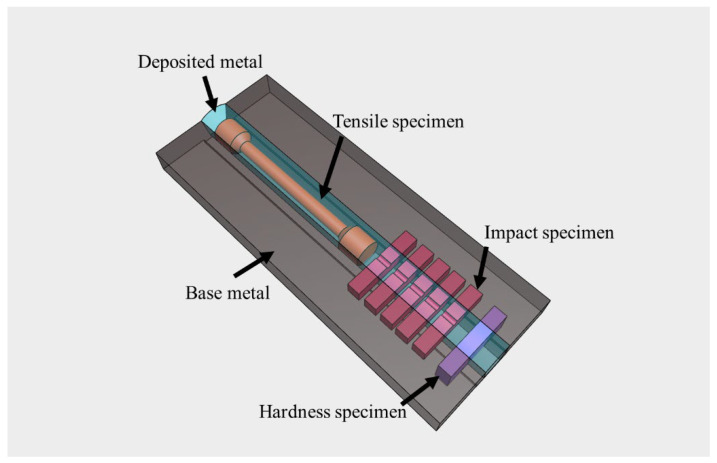
Schematic diagram of sampling for deposited metals.

**Figure 2:**
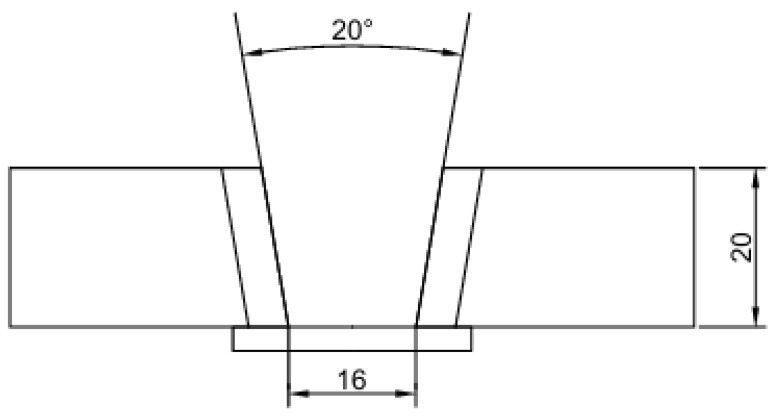
Groove schematic diagram.

Three types of electrodes were fabricated using H08E steel core wire coated with fluxes of varying niobium content (designated 0 Nb, 0.05 Nb, and 0.1 Nb), as detailed in Table 1. Q355B steel plates measuring 400 × 125 × 20 mm were selected as the base metal for deposited metal preparation, as illustrated in Figure 1. The joint configuration featured a root gap of 16 mm and a total groove angle of 20°, as shown in Figure 2. Welding was conducted in the flat position using shielded metal arc welding (SMAW) with the following parameters: welding current of 150 A, voltage of 23 V, travel speed of 120–130 mm/min, heat input of 16.5 kJ/cm, and interpass temperature of 103–107 °C. A seven-layer two-pass deposition sequence was employed, ultimately yielding three deposited metals with different Nb contents, as presented in Table 2.

### 2.2. Microstructural Characterization and Mechanical Property Tests

Vickers hardness testing was conducted on the central regions of deposited metals with varying Nb contents. The test specimens were wet-ground and polished to a mirror finish. For each specimen, five indentations were performed to obtain an average hardness value. A KB 30 S automatic Vickers hardness tester (KB Prüftechnik GmbH, Hofheim, Germany) was utilized with an applied load of 500 g and a dwell time of 15 s.

Three tensile specimens were extracted along the welding direction from each deposited metal composition for tensile testing. Tensile tests were conducted at room temperature using a CMT 5205 microcomputer-controlled electronic universal testing machine (Wanchen, Shandong, China) at a crosshead speed of 1 mm/min. The tensile specimens were rod-shaped. For each experimental group, five Charpy impact specimens were prepared and tested in accordance with ASME SFA-5.5/SFA-5.5M [19] ASME SFA/AWS standards. The impact tests were conducted at −40 °C and −60 °C using a ZBC 2302-D instrumented impact testing machine (TS, Shenzhen, China) for metallic materials.

Metallographic specimens were sectioned along the welding direction using wire electrical discharge machining (EDM). The specimens were wet-ground and polished, then etched with 4% nital for 10–15 s. After etching, the samples were rinsed with water and ethanol. The microstructure of the dried specimens was observed under a Zeiss optical microscope (Axio Observer.3m Carl Zeiss, Oberkochen, Germany). The average austenite grain size was calculated from metallographic micrographs using the line intercept method via ImageJ 7.0 software.

Phase identification was conducted via X-ray diffraction (XRD) with Cu Kα radiation on a Rigaku Smart Lab diffractometer (D8 Advance A25, Bruker, Karlsruhe, Germany) at a scanning rate of 5°/min. Diffraction patterns were acquired over a 2θ range of 10–90°.

The microstructure and precipitates of the deposited metal were analyzed by scanning electron microscopy (Zeiss Merlin Compact, Carl Zeiss, Oberkochen, Germany), transmission electron microscopy (JEM-2100F, JEOL, Tokyo, Japan), and energy dispersive spectroscopy (Zeiss Merlin Compact, Carl Zeiss, Oberkochen, Germany). Additionally, tensile and impact fracture surfaces were examined using SEM. A Zeiss Merlin Compact field emission scanning electron microscope (Zeiss Merlin Compact, Carl Zeiss, Oberkochen, Germany) was utilized. For precipitate observation and analysis, specimens underwent wet grinding and polishing procedures analogous to metallographic sample preparation, without etching. To prevent oxidation and contamination, fracture surfaces were sealed after tensile testing prior to morphological observation. All specimens were observed via SEM within 7 days. The TEM was operated at an accelerating voltage of 200 kV, with a point resolution of 0.24 nm and a line resolution of 0.14 nm.

Electron backscatter diffraction (EBSD) analysis was performed on a field-emission gun high-resolution scanning electron microscope (GAIA 3 2016, Carl Zeiss, Oberkochen, Germany) equipped with an Oxford Instruments Nordlys EBSD detector (NordlysMax2, Oxford, Wycombe, UK) to characterize the microstructure of the deposited metal. EBSD data were acquired at an accelerating voltage of 20 kV, a probe current of 20 mA, and a step size of 0.16 µm, and analyzed using EBSD 15 software.

## 3. Results

### 3.1. Microstructural Characterization of Deposited Metal

**Figure 3:**
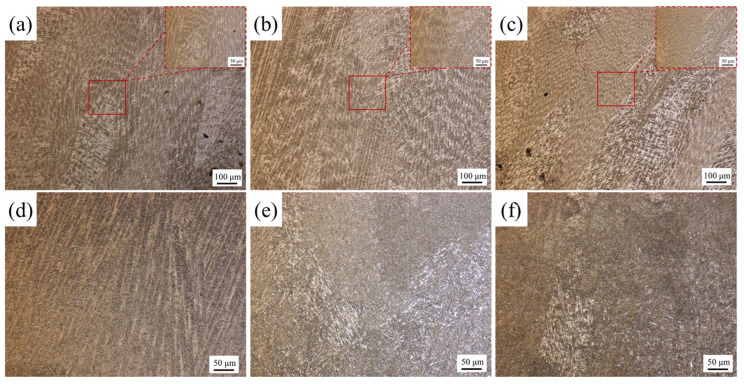
OM images of the deposited metal: (**a**–**c**) are the uppermost weld beads; (**d**–**f**) are the deposited metal; (**a**,**d**) 0 Nb; (**b**,**e**) 0.05 Nb; (**c**,**f**) 0.1 Nb.

**Figure 4:**
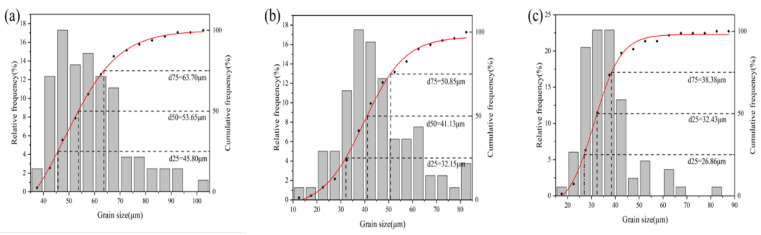
Effect of Nb Content on Lath Martensite Size: (**a**) 0 Nb; (**b**) 0.05 Nb; (**c**) 0.1 Nb.

Photographs of the metallographic organization of the deposited metal of the three covered electrodes with increasing Nb content are shown in Figure 3. The results indicate that in the uppermost weld beads of the three deposited metals (Figure 3a–c), which were not subjected to thermal influence from subsequent multipass welding, the microstructure comprises lath martensite, bainite, and second-phase particles. The micrographs reveal pronounced differences in lath martensite size. Statistical analysis of the three groups of lath martensite sizes was conducted using the line intercept method, with results presented in Figure 4. It demonstrates that as Nb content in the deposited metal increases from 0 wt% to 0.1 wt%, the lath martensite size is significantly refined, yielding average grain sizes of 53.65 μm, 41.13 μm, and 32.43 μm, respectively.

**Figure 5:**
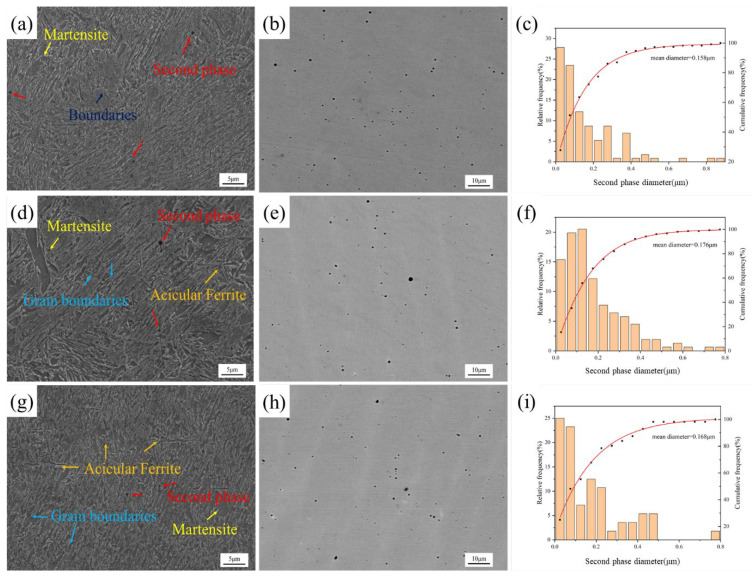
SEM images of the deposited metal: (**a**–**c**) 0 Nb; (**d**–**f**) 0.05 Nb; (**g**–**i**) 0.1 Nb.

**Figure 6:**
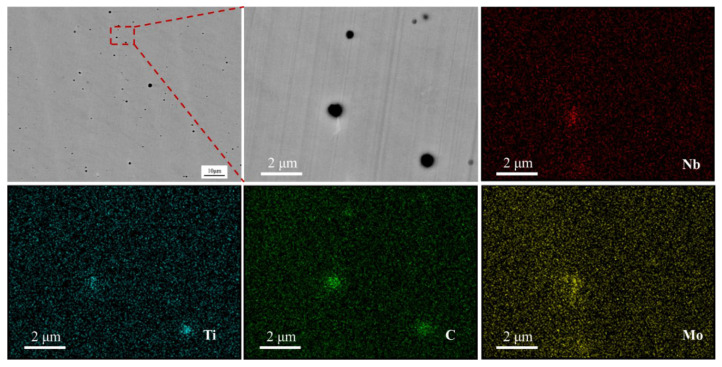
EDS mapping results of the second phase in the deposited metal.

In the central region of the deposited metal (Figure 3d–f), the microstructure transformed from austenite to tempered martensite under the influence of multiple thermal cycles during multipass welding. As observed by SEM, the microstructures in the central regions of the three deposited metals primarily consisted of tempered martensite, bainite, and second-phase particles, as shown in Figure 5a,d,g. With increasing Nb content, acicular ferrite (AF) exhibiting radial distribution and granular bainite appeared within the deposited metal; when the Nb content reached 0.1 wt%, the amount of acicular ferrite (AF) in the microstructure increased significantly. Numerous second-phase particles were clearly observed to be dispersedly distributed in the deposited metal in the unetched condition (Figure 5b,e,h). Statistical identification and analysis of a large number of second-phase particles were performed using ImageJ 7.0 software, with results presented in Figure 5c,f,i. The results indicated that the average second-phase particle sizes in deposited metals with different Nb contents were 0.158 μm, 0.176 μm, and 0.168 μm, respectively, with volume fractions of 5.69%, 5.82%, and 5.90%. The second-phase particle size was approximately 0.17 μm, showing minimal variation; however, the volume fraction of second-phase particles exhibited an increasing trend. Second-phase particles distributed in the deposited metal were characterized via EDS mapping, as shown in Figure 6. The results revealed that the precipitates were enriched in Nb, Ti, Mo, and C elements. Qi et al. [16] investigated the effect of Nb content on NiCrMo welded joints and identified that both intragranular and grain boundary precipitates were NbC phases. Chen et al. [20] studied the strengthening mechanism of Nb in high-strength hot-stamped steel and reported that the precipitates were Ti-rich(Nb, Ti)C. Wang et al. [15] demonstrated in their research on the effects of Nb and heat input on the HAZ of HSLA steel that the circular precipitated particles were Nb/Ti combined precipitates, namely, (Nb, Ti)C. Ma et al. [21] indicated in their study on Nb-Ti microalloyed steel that the precipitated particles in the steel were TiN-NbC composite precipitates. Based on the aforementioned evidence, the circular precipitates in the deposited metal are primarily composite precipitates of NbC and TiC, namely, (Nb, Ti)C.

**Figure 7:**
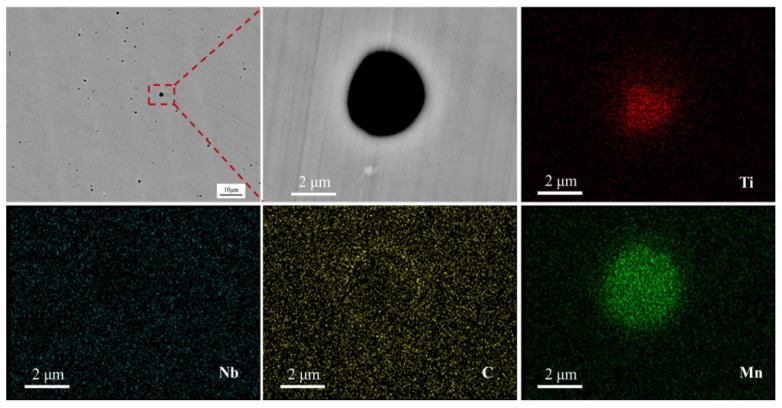
EDS mapping results of inclusions in the deposited metal.

**Figure 8:**
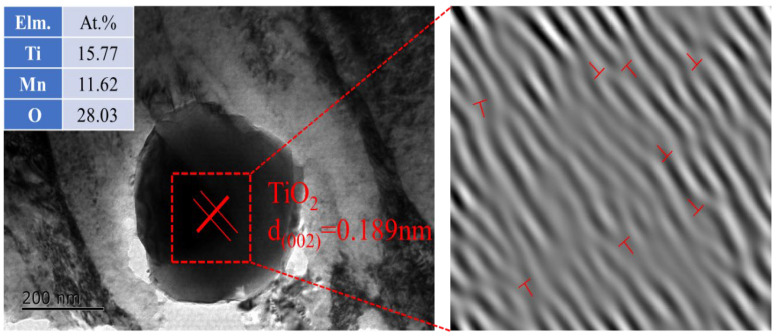
TEM and IFFT images of inclusions and dislocations in the IFFT image.

Second-phase particles with distinctly larger sizes were also observed in the deposited metal and characterized by EDS mapping, with results presented in Figure 7. EDS analysis revealed that these particles were enriched in Ti and Mn but devoid of Nb and C, suggesting they constitute inclusions of a distinct composition. To further investigate the nature and influence of these inclusions, transmission electron microscopy (TEM) was performed on the 0.05 Nb deposited metal, with results shown in Figure 8. TEM observations indicated that the inclusion exhibited a size of approximately 0.4 μm and was enriched in Ti and O. The inverse fast Fourier transform (IFFT) image revealed substantial dislocation pile-ups in the vicinity of the inclusion, with an interplanar spacing of d(002) = 0.189 nm. By comparison with the standard d(220) interplanar spacing of TiO_2_ (0.1957 nm), the inclusion was identified as a TiO_2_ inclusion, which likely originated from slag-forming or arc-stabilizing agents in the electrode coating.

In summary, the second-phase particles in the deposited metal primarily consist of composite (Nb, Ti)C precipitates and TiO_2_ inclusions.

**Figure 9:**
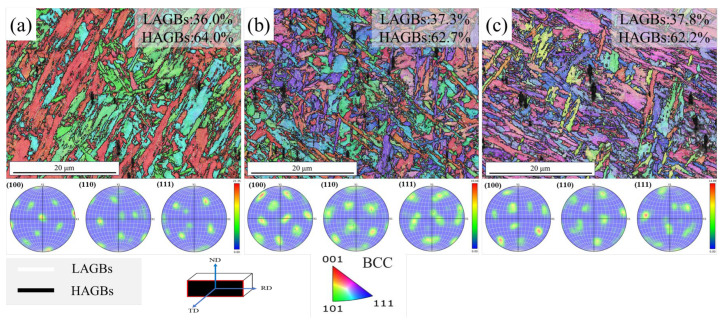
EBSD images and pole figures of the deposited metal: (**a**) 0 Nb; (**b**) 0.05 Nb; (**c**) 0.1 Nb.

To investigate the effects of Nb content on the microstructure and crystallographic orientation of deposited metals, electron backscatter diffraction (EBSD) analysis was performed on three deposited metals with varying Nb contents, as presented in Figure 9. The results indicate that the microstructures of all three deposited metals are predominantly composed of lath martensite, with a decreasing trend observed in the grain size of lath martensite. White and black lines in the figures represent low-angle grain boundaries (LAGBs) and high-angle grain boundaries (HAGBs), respectively. Figure 9 shows that the fraction of HAGBs in deposited metals with different Nb contents remains between 62% and 64%.

Regarding grain morphology, the 0 Nb deposited metal predominantly exhibited elongated, fibrous structures characterized by continuous, color-gradient patterns, with relatively large grain sizes and orientations primarily along the [001] and [101] directions. With increasing Nb content, fine equiaxed grains began to appear at 0.05 Nb, accompanied by significant grain refinement and an orientation shift toward the [111] direction. At 0.1 Nb, the microstructure displayed uniform equiaxed grains with more pronounced refinement, predominantly oriented in the [111] direction. Pole figure analysis revealed that the 0 Nb sample exhibited diffusely distributed spots across all directions, indicating the presence of a certain texture with a maximum intensity of 21.35 and high orientation density. With Nb addition, the maximum texture intensity decreased to 10.06 at 0.05 Nb and 14.89 at 0.1 Nb. This demonstrates that Nb addition weakens the original texture, although higher Nb content may slightly increase texture intensity. Overall, Nb addition is beneficial for improving the mechanical properties of the deposited metal.

**Figure 10:**
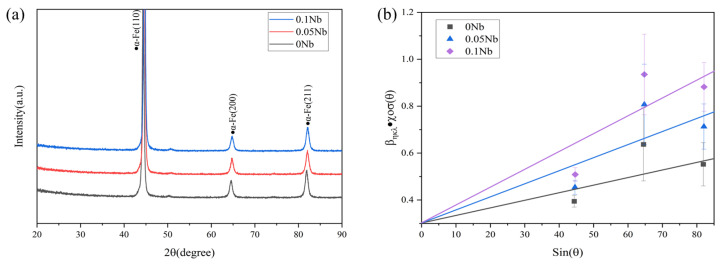
(**a**) Examples of the measured XRD profiles; (**b**) Williamson–Hall plots of diffraction patterns for different Nb contents.

The phase composition of the deposited metal was analyzed by X-ray diffraction (XRD), with results shown in Figure 10a. The α-Fe phase (PDF#85-1410) was observed as the primary constituent. Dislocation density was calculated from the XRD patterns, assuming internal strain was solely generated by dislocations. Three prominent diffraction peaks were selected, and the Williamson–Hall method [22] was employed to determine the dislocation density in martensite, with data analysis performed using Jade 6.0 software. Dislocation density (ρ) was calculated using the following formula [23]:

(1) $$\rho =k\frac{\varepsilon^{2}}{b^{2}}$$

In the equation, b is the Burgers vector (0.2482 nm) [24] and k is a geometric constant. Since α-Fe is a body-centered cubic (BCC) structured material, k = 1.44 [23].

Studies have demonstrated [22,23] that peak broadening originates primarily from lattice strain (ε) alone, or from the combined contributions of lattice strain and small crystallite size, with both mechanisms yielding similar Cauchy profiles. The lattice strain can be estimated using the following formula [22]:

(2) $$\beta_{hkl}=\frac{\lambda }{Dcos\theta }+2\varepsilon \;tan\theta \to \beta_{hkl}\;cos\theta =\frac{\lambda }{D}+2\varepsilon \;tan\theta$$

In the equation, *θ* is the Bragg angle and $\beta_{hkl}$ is the spectral integral line breadth of the diffraction peak. The broadening of the instrument can be eliminated by detecting a standard corundum specimen, and is interpolated for the angular peak position *θ*. In this study, all specimens were fully crystallized and exhibited no fracture during processing; therefore, the contribution of crystallite size to $\beta_{hkl}$ was deemed negligible. In this study, all specimens were fully crystallized and exhibited no fracture during processing; therefore, the contribution of crystallite size to $\beta_{hkl}$ was deemed negligible.

The Williamson–Hall plots were constructed from these diffraction patterns with Sin*θ* as the x-axis and $\beta_{hkl}$·cos*θ* as the y-axis, as shown in Figure 10b, and the y-intercept was set to zero. The lattice strain ε of the samples was obtained from the slope of the fitted line, yielding values of 0.253%, 0.274%, and 0.289%, respectively. The calculated dislocation densities of martensite in the three deposited metals with different Nb contents were 1.5 × 10^15^ m^−2^, 1.75 × 10^15^ m^−2^, and 1.95 × 10^15^ m^−2^, respectively.

### 3.2. Mechanical Properties of Deposited Metal

**Figure 11:**
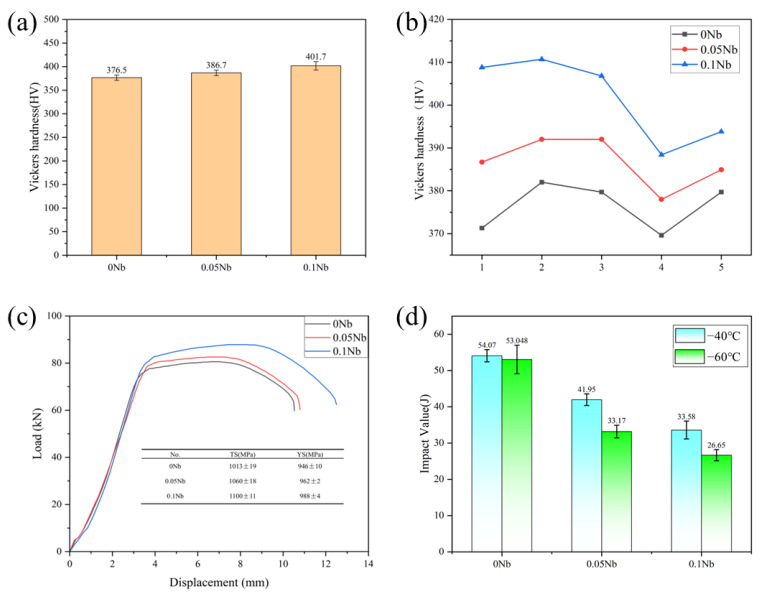
Mechanical Properties of Deposited Metal: (**a**,**b**) Vickers hardness; (**c**) Tensile strength, Yield strength, and Load–Displacement Curves; (**d**) Low-temperature impact toughness.

The Vickers hardness testing was performed on the central regions of the three deposited metal specimens with different Nb contents at room temperature. The experimental results are presented in Figure 11a. The hardness values of deposited metals with different Nb contents were 376.5 HV, 386.7 HV, and 401.7 HV, respectively, demonstrating that hardness increases with Nb content. Tensile testing was conducted at room temperature on the three deposited metals with different Nb contents, and Charpy impact tests were performed on U-notched impact specimens at −40 °C and −60 °C. The tensile testing and low-temperature impact results are shown in Figure 11c,d. At room temperature, the 0 Nb specimen exhibited a tensile strength of 1013 MPa and a yield strength of 946 MPa, with mean impact energies of 54.07 J and 53.048 J at −40 °C and −60 °C, respectively, representing the highest low-temperature impact toughness among the three groups, which showed minimal variation with decreasing temperature, but the lowest strength. The 0.05 Nb specimen demonstrated a tensile strength of 1060 MPa and a yield strength of 962 MPa at room temperature, with mean impact energies of 41.95 J and 33.17 J at −40 °C and −60 °C, respectively. The 0.1 Nb specimen exhibited a tensile strength of 1100 MPa and a yield strength of 988 MPa at room temperature, with mean impact energies of 33.58 J and 26.65 J at −40 °C and −60 °C, respectively, representing the lowest low-temperature impact toughness but the highest strength among the three groups. The data demonstrate that low-temperature impact toughness is inversely proportional to Nb content, decreasing significantly with increasing Nb content, while hardness and strength are directly proportional to Nb content, increasing progressively with Nb addition.

## 4. Discussion

### 4.1. Effect of Nb Content on the Microstructure of Weld Metal

Since the welding process parameters employed for preparing the three deposited metals were identical, and only the Nb concentration was varied in the alloy design, while other alloying elements, impurity elements, and inclusions remained at equivalent concentrations, the microstructural evolution of the weld metal was entirely governed by the variation in Nb concentration. As shown in Figure 5, increasing Nb content promoted the precipitation of (Nb, Ti)C, leading to an increased volume fraction of second-phase particles in the deposited metal. These (Nb, Ti)C precipitates exerted a dragging force at grain boundaries, impeding their mobility and thereby pinning the grain boundaries to suppress grain growth, ultimately achieving grain refinement and strengthening of the deposited metal. Furthermore, both (Nb, Ti)C precipitates and TiO_2_ inclusions can serve as nucleation sites within the deposited metal, inducing the formation of acicular ferrite (AF); consequently, the proportion of acicular ferrite increased with rising Nb content.

### 4.2. Effect of Nb Content on the Mechanical Properties of Weld Metal

Vickers hardness testing was conducted at identical locations across the three deposited metals, with results presented in Figure 11b. A distinct hardness reduction was observed at position 4 for all three groups, followed by a recovery at position 5. This hardness variation is attributable to the heterogeneous microstructural constituents within the deposited metal, which comprises relatively hard martensite and comparatively soft bainite. Position 4 was situated within the bainite region, thereby accounting for the observed hardness decrease.

**Figure 12:**
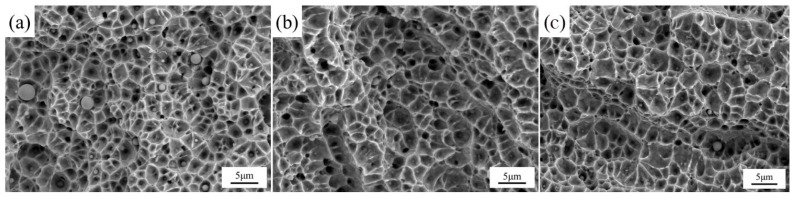
SEM images of the tensile fracture surfaces: (**a**) 0 Nb; (**b**) 0.05 Nb; (**c**) 0.1 Nb.

Microstructural characterization was performed on the tensile and impact fracture surfaces of deposited metals with three Nb contents. Figure 12a–c present SEM micrographs of the tensile fracture surfaces. All three deposited metals exhibited numerous dimples on the fracture surfaces, characteristic of a typical ductile fracture mode. A substantial quantity of second-phase particles was also observed within the dimples. Based on the preceding analysis, these second-phase particles were predominantly relatively large TiO_2_ inclusions. These inclusions promote the formation of acicular ferrite, impede dislocation motion, and refine grain size, thereby further contributing to the enhancement of deposited metal strength.

**Figure 13:**
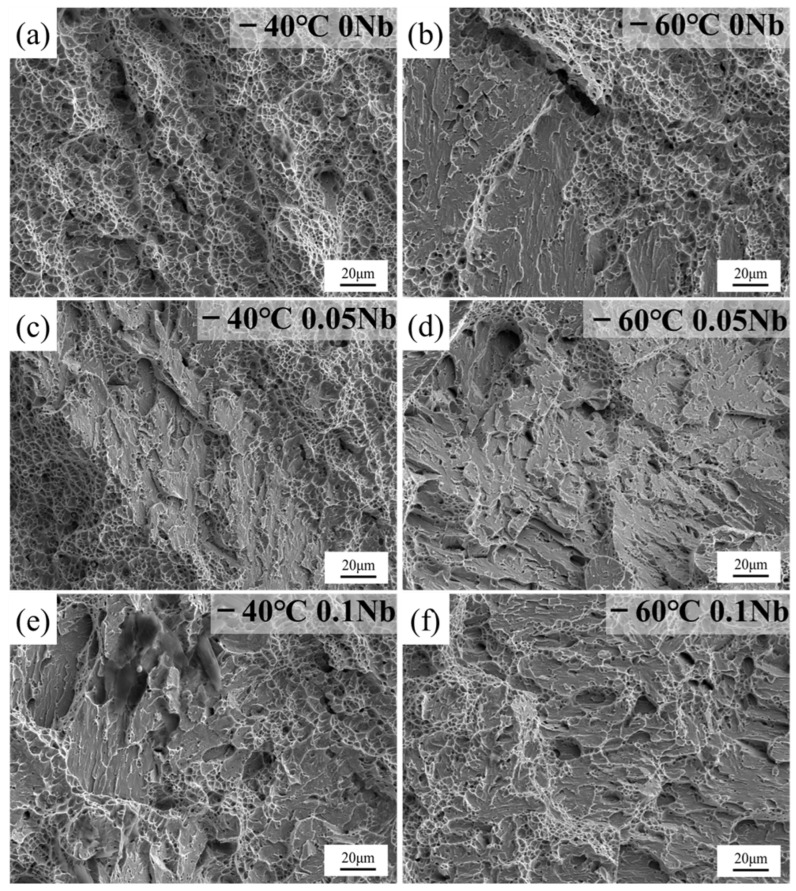
SEM images of the impact fracture surfaces: (**a**,**b**) 0 Nb; (**c**,**d**) 0.05 Nb; (**e**,**f**) 0.1 Nb; (**a**,**c**,**e**) −40 °C impact fracture; (**b**,**d**,**f**) −60 °C impact fracture.

**Figure 14:**
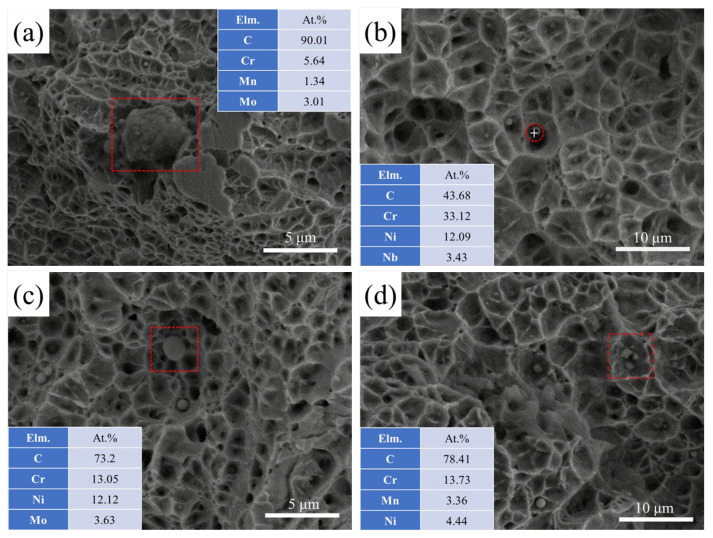
EDS mapping results of precipitates in impact fracture surfaces.

Figure 13 illustrates the impact fracture surfaces of the three deposited metal groups at different temperatures. SEM observations reveal that the 0 Nb specimen exhibits abundant dimples at −40 °C, indicating a predominantly ductile fracture mode. With increasing Nb content, the number of dimples decreases while river-pattern cleavage facets progressively increase, resulting in a fracture mechanism transition from ductile to quasi-cleavage fracture. At −60 °C, the proportion of river-pattern cleavage facets on the fracture surface is larger compared to that at −40 °C. Under high magnification, precipitates can be observed within the dimples on the fracture surfaces. EDS analysis of these precipitates is presented in Figure 14. The results demonstrate that the precipitates are enriched in C, Cr, Mo, Ni, and Mn elements, suggesting they may be MC-type, M_23_C_6_-type, and M_6_C-type carbides or other complex inclusions. Specifically, Nb-rich precipitation is observed in the precipitates shown in Figure 14b, while the Mn element appears in Figure 14a,d. These findings align with the theories proposed by Qi [16] and Mohammed Ali [17], who suggested that Nb-rich phases exhibit brittleness during low-temperature impact testing and that excessive precipitation of Nb-rich phases adversely affects low-temperature impact toughness. Furthermore, Nb promotes the precipitation of Mn-containing complex inclusions, which represents a critical factor in toughness degradation. This phenomenon of Nb-rich precipitation and Mn-containing complex inclusions may be the primary cause of the observed reduction in impact toughness.

### 4.3. Strengthening Effects

#### 4.3.1. Grain Refining Strengthening

According to the Hall–Petch equation, the grain refinement strengthening effect can be expressed as:

(3) $$\sigma_{g}\;=k_{y}d^{-\frac{1}{2}}$$

In the equation, *d* is the effective grain size, $k_{y}$ is the Hall–Petch coefficient. Chen et al. [20] proposed that the yield strength of tempered martensitic steel is proportional to the inverse square root of tempered martensite lath width, with a Hall–Petch coefficient of 120 MPa μm^1/2^. Morito et al. [25] suggested that block size is the critical microstructural parameter when analyzing the strength-microstructure relationship in low-carbon steel lath martensite. Therefore, the average width of martensite blocks was employed as the effective grain size. By statistically analyzing the average width of martensite blocks in Figure 9 as the effective grain size, the martensite grain sizes for the three deposited metals were determined to be 0.78 μm, 0.75 μm, and 0.58 μm, respectively. Calculation results indicated that the grain refinement strengthening contributions for deposited metals with the three Nb contents were 135.5 MPa, 138.5 MPa, and 157.6 MPa, respectively.

#### 4.3.2. Dislocation Strengthening

HajyAkbary F et al. [26] calculated the dislocation density of martensite using X-ray diffraction line profile analysis, which showed excellent agreement with values determined from dislocation strengthening. The dislocation strengthening of the martensite can be expressed as follows [27]:

(4) $$\sigma_{d}\;=M\alpha Gb\rho^{\frac{1}{2}}$$

In the equation, M is the Taylor factor, taken as M = 3 [28], α is a constant at 0.24, b is the magnitude of the Burgers vector (0.2482 [24]). Ghos et al. [29] identified a lath structure with higher dislocation density compared to its cubic ferrite counterpart, exhibiting a shear modulus of 76 GPa. J. M. Oblak et al. [30] similarly adopted a shear modulus of 76 GPa for Ni-based alloys. Substituting the calculated dislocation density values and relevant parameters into Equation (4) yielded dislocation strengthening contributions of 526 MPa, 568 MPa, and 600 MPa for the deposited metals with the three Nb contents, respectively.

#### 4.3.3. Solid Solution Strengthening

**Figure 15:**
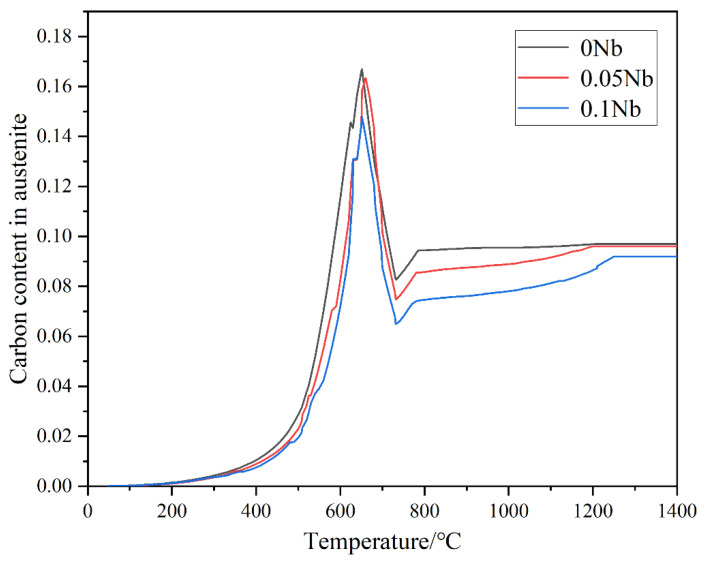
Carbon content in austenite.

According to the study by Yong [31], solid solution strengthening contribution can be expressed by the following equation:

(5) $$\sigma_{s}=4570\left[C\right]+83\left[Si\right]+37\left[Mn\right]+40\left[Mo\right]+80\left[Ti\right]+470\left[P\right]-30\left[Cr\right]$$

where [X] represents the weight fraction of element X in solution (wt%). Except for carbon, the weight fractions of other elements in the deposited metal are essentially equivalent to their concentrations in solid solution, as carbon is partially consumed by precipitated carbides during the solid solution process. The solid solution carbon content in austenite at various temperatures was calculated using Jmatpro7.0 software, with results presented in Figure 15. As temperature decreases, the carbon content in austenite progressively diminishes, approaching essentially zero at room temperature (25 °C). Given that Nb and Ti in the deposited metal primarily precipitate as carbonitrides, and the overall carbon content is low, being largely consumed by Nb and Ti to form carbides, the solid solution concentration of carbon is effectively negligible. Simultaneously, Nb and Ti exist predominantly in carbide form with minimal solid solution content, rendering their contributions to solid solution strengthening almost zero. Substituting the remaining elements (Si, Mn, Mo, P, Cr) into Equation (5) yielded solid solution strengthening values of 129.3 MPa, 129.0 MPa, and 131.6 MPa for the three deposited metals with varying Nb contents.

#### 4.3.4. Precipitation Strengthening

The precipitation strengthening contribution can be quantitatively calculated using the Ashby–Orowan equation [32,33] under the assumption of a precipitate bypassing mechanism [34], as expressed by the following formula:

(6) $$\sigma_{p}=\left(\frac{0.528Gb\sqrt{V_{f}}}{X}\right)\ln\left(\frac{X}{2b}\right)$$

In the equation, G represents the shear modulus (G = 76 GPa), b is the Burgers vector (b = 0.2482 nm), and X and $V_{f}\;$ denote the average precipitate diameter and precipitate volume fraction, respectively. Substituting the relevant parameters into the Equation (6) yielded precipitation strengthening values of 86.7 MPa, 80.1 MPa, and 82.5 MPa for the three deposited metals with varying Nb contents.

#### 4.3.5. Discussion of Calculation Results

**Figure 16:**
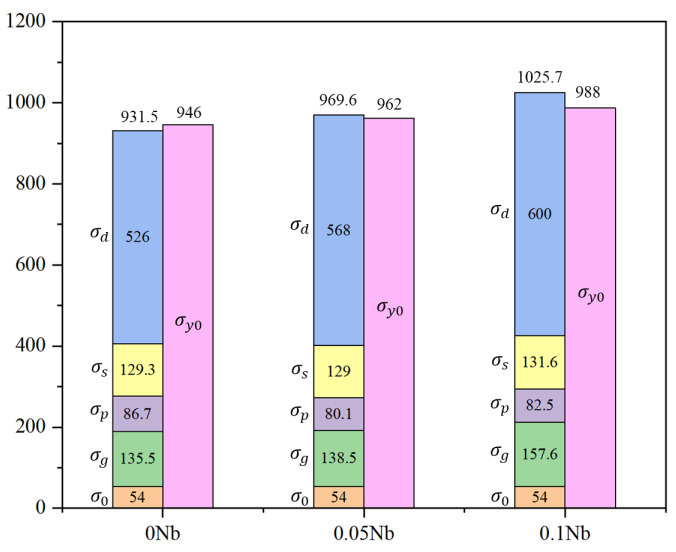
Proportional contributions of strengthening mechanisms.

In this study, the yield strength of lath martensite ($\sigma_{y}$) was assumed to result from the simple additive contributions of various strengthening mechanisms; accordingly, the yield strength values of the deposited metal were calculated using the following formula [35]:

(7) $$\sigma_{y}=\sigma_{0}+\sigma_{g}+\sigma_{d}+\sigma_{s}+\sigma_{p}$$

where $\sigma_{0}$ is the lattice friction stress for pure Fe ($\sigma_{0}$ = 54 MPa), $\sigma_{g}$, $\sigma_{d}$, $\sigma_{s}$, and $\sigma_{p}\;$ are the strengthening effects caused by grain refinement, dislocation, solid solution, and precipitation, respectively. The calculated yield strength values for deposited metals with different Nb contents are presented in Table 3. The calculated yield strengths show some deviation from the actual measured values, though the differences fall within the margin of error, indicating reasonable accuracy. The results demonstrate that dislocation strengthening in martensite constitutes the dominant strengthening mechanism in 960 MPa grade HSLA steel, accounting for 56–60% of the total yield strength, as shown in Figure 16. As Nb content increases, the dislocation strengthening effect in martensite rises from 526 MPa to 600 MPa; grain refinement strengthening increases from 135.5 MPa to 157.6 MPa, exhibiting an upward trend as Nb addition refines the grain structure of the deposited metal. Nb addition has minimal influence on solid solution strengthening and precipitation strengthening, with no significant variation observed in these contributions across different Nb contents.

## 5. Conclusions

In this study, three HSLA steel deposited metals with different Nb contents were fabricated by adjusting the electrode coating composition and employing the shielded metal arc welding (SMAW) method, leading to the following conclusions:

(1) Under identical welding conditions, the dominant microstructure transformed from martensite to tempered martensite due to multipass welding thermal cycles, with minor amounts of bainite and second-phase particles. The grain size of the deposited metal was significantly refined as Nb content increased from 0 wt% to 0.1 wt%.
(2) As Nb content increases, the hardness and strength of the deposited metal progressively increase, while the low-temperature impact toughness correspondingly decreases. The reduction in impact toughness is primarily attributed to Nb-rich precipitates and Mn-containing complex inclusions. Among the tested compositions, the deposited metal containing 0.05 wt% Nb exhibits the optimal comprehensive mechanical properties.
(3) With increasing Nb content, the dislocation strengthening effect in martensite increased from 526 MPa to 600 MPa, which is the most dominant strengthening mechanism; the grain refinement strengthening effect increased from 135.5 MPa to 157.6 MPa. Nb addition exhibited negligible influence on solid solution and precipitation strengthening.

## Figures and Tables

**Table 1:** Chemical composition of H08E steel core wire (wt%).

CW	C	Si	Mn	Cr	Mo	Ni	Cu	N	Al	Fe
H08E	0.094	0.06	0.52	0.05	0.01	0.03	0.05	0.005	0.001	Bal.

**Table 2:** Chemical composition of the deposited metals (wt%).

No.	C	Nb	Si	Mn	Cr	Mo	Ni	Ti	V	P	S
0 Nb	0.097	0.001	0.39	1.79	0.34	0.92	4.29	0.016	0.009	0.006	0.004
0.05 Nb	0.096	0.047	0.36	1.77	0.35	0.93	4.26	0.013	0.009	0.006	0.004
0.1 Nb	0.092	0.097	0.39	1.82	0.35	0.92	4.26	0.015	0.009	0.006	0.004

**Table 3:** Dissociated strength contribution from various strengthening mechanisms (MPa).

No.	*σ*_y_	*σ*_0_	*σ*_g_	*σ*_d_	*σ*_s_	*σ*_p_
0 Nb	931.5	54	135.5	526	129.3	86.7
0.05 Nb	969.6	54	138.5	568	129.0	80.1
0.1 Nb	1025.7	54	157.6	600	131.6	82.5

Notes: $\sigma_{y}$, $\sigma_{0}$,$\;\sigma_{g}$,$\;\sigma_{d}$, $\sigma_{s}$, and $\sigma_{p}$ represent yield strength, the lattice friction stress of pure iron, grain refinement strengthening, dislocation strengthening, solid solution strengthening, and precipitation strengthening, respectively.

## Data Availability

The original contributions presented in this study are included in the article. Further inquiries can be directed to the corresponding authors.
